# Androgen-sensitive hypertension associated with soluble guanylate cyclase alpha1 deficiency is mediated by 20-HETE

**DOI:** 10.1186/2050-6511-16-S1-A97

**Published:** 2015-09-02

**Authors:** Sara Vandenwijngaert, Ana C Dordea, Victor Garcia, Robert E Tainsh, Daniel I Nathan, Michael J Raher, Kaitlin Allen, Fan Zhang, Wolfgang S Lieb, Sarah Mikelman, Andrew Kirby, Christine Stevens, Robrecht Thoonen, Allyson Hindle, Patrick Y Sips, Rajeev Malhotra, Mark J Daly, Peter Brouckaert, Kenneth D Bloch, Michal Schwartzman, Emmanuel S Buys

**Affiliations:** 1 Department of Anesthesia, Critical Care, and Pain Medicine, Massachusetts General Hospital and Harvard Medical School, Boston, MA 02114, USA; 2 Department of Pharmacology, New York Medical College, Valhalla, NY 10595, USA; 3 Center for Human Genetic Research, Department of Medicine, Massachusetts General Hospital, Harvard Medical School, Boston, MA 02114, USA; 4 Division of Cardiovascular Medicine, Department of Medicine, Brigham and Women's Hospital, Boston, MA 02115, USA; 5 Department for Biomedical Molecular Biology, Ghent University, Ghent, 9000, Belgium

## Background

Dysregulated nitric oxide (NO) signaling contributes to the pathogenesis of hypertension. Previously, we reported gender- and strain-specific hypertension in mice deficient in the α1-subunit of the NO receptor soluble guanylate cyclase (sGCα1^-/-^): male mice on an Sv129/J (S6) but not a C57BL6/J (B6) background are hypertensive.

## Methods and results

Via linkage analysis, we identified a quantitative trait locus (QTL) associated with elevated blood pressure in male sGCα1^-/-^S6 mice. This QTL encompasses *CYP4a12a*, encoding the predominant murine synthase of the vasoconstrictor 20-hydroxyeicosatetraenoic acid (20-HETE). Renal expression of *CYP4a12a*was strain-, gender-, and testosterone-dependent:*CYP4a12a* gene expression was higher in male WT and sGCα_1_^-/-^S6 mice than in female S6 mice, or than in male and female, WT and sGCα_1_^-/-^ B6 mice, higher in testosterone-treated S6 mice than in vehicle-treated S6 mice, and higher in sham-operated S6 mice than orchiectomized S6 mice. Also, 20-HETE levels were higher in renal preglomerular microvessels of male sGCα1^-/-^ S6 than of sGCα1^-/-^ B6 mice. Furthermore, the 20-HETE antagonist 20-6,15-HEDGE lowered blood pressure in male sGCα_1_^-/-^ S6 but not WT mice. Finally, the more significant impairment of acetylcholine-induced relaxation of renal interlobar arteries in male sGCα_1_^-/-^ S6 than sGCα_1_^-/-^ B6 mice, in male sGCα_1_^-/-^ S6 than WT S6 mice, and in male sham-operated sGCα_1_^-/-^ S6 mice than orchiectomized sGCα_1_^-/-^ S6 mice was rescued by 20-6,15-HEDGE.

## Conclusion

Gender- and strain-specific hypertension and vascular dysfunction in sGCα1^-/-^ S6 mice is associated with elevated *CYP4a12a* expression and 20-HETE levels, and is abrogated by antagonizing 20-HETE. These results corroborate our hypothesis that testosterone-induced *CYP4a12a* expression and a concomitant increase in 20-HETE production contribute to the hypertension associated with impaired NO-cGMP signaling and that C*YP4a12a* represents a candidate blood pressure modifying gene in the context of deficient NO-sGC signaling.

